# INPP5D/SHIP1 in Alzheimer’s Disease: Linking Microglial Substrate Handling to Neuroinflammation and Neurovascular Dysfunction

**DOI:** 10.3390/neurolint18100186

**Published:** 2026-09-29

**Authors:** Junxiang Xu, Yijin Chang, Dong Liu, Changsheng Chen

**Affiliations:** School of Life Sciences, Co-Innovation Center of Neuroregeneration, Nantong University, Nantong 226019, China

**Keywords:** INPP5D, SHIP1, Alzheimer’s disease, microglia, neuroinflammation, neurovascular dysfunction

## Abstract

INPP5D, which encodes Src homology 2 domain-containing inositol 5-phosphatase 1 (SHIP1), has emerged as an Alzheimer’s disease (AD)-associated gene with strong links to microglial biology. As a hematopoietic-enriched lipid phosphatase, SHIP1 converts phosphatidylinositol-3,4,5-trisphosphate to phosphatidylinositol-3,4-bisphosphate, thereby regulating phosphoinositide-dependent receptor signaling, membrane remodeling, phagocytosis, vesicle trafficking, and inflammatory responses. In AD, microglia are chronically exposed to amyloid-β, lipid-rich debris, damaged synapses, complement-tagged structures, and inflammatory mediators. These substrates require coordinated uptake, endolysosomal processing, autophagic adaptation, and controlled inflammatory output. Current data place INPP5D/SHIP1 at a step after receptor engagement, where microglial uptake must be coupled to vesicular routing and lysosomal degradation. When this coupling fails, engulfed amyloid, lipid debris, or synaptic material may accumulate in stressed endolysosomal compartments, promoting defective autophagy, NLRP3 inflammasome activation, and sustained cytokine release. Although current data support a primarily microglial or myeloid-centered role for INPP5D in the brain, altered microglial states may secondarily affect the neurovascular unit through cytokine release, complement activation, oxidative stress, and impaired amyloid or lipid clearance. This review summarizes the molecular function of INPP5D/SHIP1 in AD-related microglial signaling and discusses its implications for phagocytosis, endolysosomal stress, inflammasome activation, therapeutic targeting, and microglia–vascular communication.

## 1. Introduction

Alzheimer’s disease (AD) is defined neuropathologically by amyloid-β deposition, tau aggregation, synaptic degeneration, and progressive neuronal loss. These lesions remain central to diagnosis and staging, but they do not fully explain the genetic and cellular heterogeneity of the disease. Large-scale genetic studies have repeatedly implicated immune, lipid-processing, and endocytic pathways in AD susceptibility [1,2,3,4,5]. Several AD risk genes, including *TREM2*, *PLCG2*, *ABI3*, *CD33*, *ABCA7*, and *INPP5D*, are preferentially expressed in microglia or other myeloid-lineage cells, shifting attention from neurons alone to the cellular systems that regulate amyloid handling, lipid metabolism, and inflammatory remodeling [6,7,8]. This genetic architecture places microglia at a central point for understanding how innate immune signaling modifies AD progression.

INPP5D encodes Src homology 2 domain-containing inositol 5-phosphatase 1 (SHIP1), a hematopoietic-enriched lipid phosphatase positioned downstream of PI3K signaling. SHIP1 dephosphorylates phosphatidylinositol-3,4,5-trisphosphate to generate phosphatidylinositol-3,4-bisphosphate, thereby altering membrane-associated signaling events that regulate receptor activation, actin remodeling, endocytosis, vesicle trafficking, and lysosomal routing [9,10,11,12,13,14,15,16]. In AD, INPP5D expression and genetic variation have been associated with disease risk and plaque-associated microglial states [17,18,19,20], while experimental studies link SHIP1 activity to amyloid pathology, microglial responses to amyloid-β, TREM2-related functions, autophagy, and inflammasome activation [21,22,23,24,25]. The central mechanistic problem lies downstream of activation: after microglia engulf amyloid, lipid debris, or synaptic material, SHIP1-dependent signaling may determine whether these substrates are degraded, retained in endolysosomal compartments, or allowed to trigger inflammatory stress. TREM2-dependent programs support plaque-associated microglial responses and lipid handling [26,27,28,29,30], CD33 has been linked to impaired microglial uptake of amyloid-β [31,32], and complement signaling can drive synaptic engulfment in AD models [33,34]. Once internalized, these substrates must be processed through endosomal, lysosomal, and autophagic systems [35,36,37,38,39]. Failure at this level can promote lysosomal stress and create conditions that favor NLRP3 inflammasome activation [40,41,42].

This review discusses INPP5D/SHIP1 as a microglial lipid-signaling regulator that may influence the transition from substrate clearance to inflammatory amplification in AD. Its neurovascular relevance is considered within the same framework. Current evidence supports a predominantly microglial or myeloid-centered role for INPP5D in the brain, rather than a primary endothelial function [17,18,19,20]. The neurovascular unit is nevertheless sensitive to microglial state changes: BBB integrity depends on coordinated interactions among endothelial cells, pericytes, astrocytic endfeet, neurons, microglia, and the basement membrane [43,44,45,46], and BBB breakdown, pericyte dysfunction, altered cerebral blood flow, and impaired vascular clearance are closely associated with cognitive decline and amyloid burden in aging and AD [47,48,49]. We therefore focus on phosphoinositide signaling, microglial phagocytosis, endolysosomal stress, NLRP3 inflammasome activation, and secondary effects on the neurovascular unit.

## 2. Molecular Function of INPP5D/SHIP1 in Phosphoinositide Signaling

INPP5D encodes SHIP1, a member of the inositol polyphosphate 5-phosphatase family with predominant expression in hematopoietic and myeloid-lineage cells [9,10,11,12,17,20]. SHIP1 dephosphorylates phosphatidylinositol-3,4,5-trisphosphate [PI(3,4,5)P3 or PIP3] at the 5-position to generate phosphatidylinositol-3,4-bisphosphate [PI(3,4)P2], placing the enzyme immediately downstream of PI3K activation [9,10]. The reaction does more than reduce PIP3 abundance. It also produces PI(3,4)P2, a signaling lipid with distinct effector-binding properties [13,14]. Through this change in membrane phosphoinositide composition, SHIP1 can alter the recruitment of proteins involved in immune receptor signaling, cytoskeletal remodeling, endocytosis, vesicle trafficking, and cell survival [11,12,13,14,15,16].

This signaling position is directly relevant to microglial biology in AD. Amyloid-β, lipid-rich debris, apoptotic material, complement-opsonized synapses, and inflammatory cytokines engage receptor systems that depend on phosphoinositide remodeling and PI3K-related signaling [16,26,50,51]. In this setting, SHIP1 is better interpreted as a regulator of signal routing than as a simple inhibitor of PI3K-AKT activity. Hydrolysis of PIP3 can reduce selected AKT-dependent signals, whereas PI(3,4)P2 can support membrane-associated processes required for actin reorganization, phagocytic cup formation, substrate internalization, and endosomal trafficking [13,14,16]. Recent evidence further extends this view beyond SHIP1 phosphatase activity. The N-terminal SH2 domain of INPP5D directly recognizes phosphorylated RIPK1 and suppresses RIPK1 kinase activation, thereby restraining RIPK1-dependent inflammatory programs in microglia. Loss of INPP5D consequently enhances the expression of multiple inflammatory mediators and late-onset AD-associated genes, including components of complement, NLRP3, and gasdermin D [52]. Changes in INPP5D activity may therefore alter both the intensity and the cellular consequence of microglial activation. Together, these findings broaden the functional view of SHIP1 from a phosphoinositide phosphatase that modifies PI3K–AKT signaling to a multidomain regulator integrating lipid signaling with protein-mediated control of inflammatory pathways, potentially helping to explain the diverse phenotypes observed after SHIP1 manipulation, including changes in amyloid pathology, plaque-associated microglial responses, autophagy, inflammasome activation, and TREM2-related microglial functions [17,21,22,24,25,28,30,52].

## 3. INPP5D/SHIP1 and Microglial Phagocytosis in AD

Microglial phagocytosis has different consequences depending on the substrate being cleared and the intracellular capacity available to process it. In AD models, plaque-associated microglial programs driven in part by TREM2 support clustering around amyloid deposits and lipid handling [26,27,28,29,30,53]. CD33, in contrast, has been linked to reduced microglial uptake of amyloid-β [31,32]. Microglia also engulf apoptotic material, damaged neurites, myelin debris, and lipid-rich particles; these activities can contribute to tissue repair when degradation and metabolic adaptation are sufficient. The same machinery can become harmful when it is redirected toward complement-tagged synapses or when chronic substrate exposure overwhelms lysosomal processing [33,34,35,54]. Phagocytosis in AD should therefore be evaluated by substrate identity, disease stage, vesicular routing, and degradative outcome, rather than by uptake rate alone.

This distinction between substrate uptake and intracellular processing is supported by recent evidence involving CD22. In primary microglia, CD22 knockdown enhanced intracellular Aβ degradation without significantly altering initial Aβ uptake, indicating regulation at a post-engulfment step. CD22 depletion also reduced INPP5D expression and increased AKT phosphorylation, whereas INPP5D overexpression partially reversed the enhanced Aβ clearance induced by CD22 knockdown [55]. These findings support a functional link between CD22 and INPP5D in post-engulfment Aβ processing, although the mechanism linking CD22 to INPP5D and the contribution of altered AKT activity to intracellular Aβ degradation remain unresolved.

INPP5D/SHIP1 is linked to phagocytosis through phosphoinositide control of membrane remodeling. Formation of a phagocytic cup requires receptor activation, local actin rearrangement, membrane extension, vesicle scission, and subsequent delivery to endosomal and lysosomal compartments. By converting PIP3 to PI(3,4)P2, SHIP1 can alter signaling at both the plasma membrane and intracellular vesicles [9,10,11,13,14]. This is relevant to several AD-associated inputs, including amyloid-β, lipid debris, apoptotic cells, and complement-opsonized synapses. Experimental work supports this link: INPP5D deficiency has been reported to attenuate amyloid pathology in a mouse AD model, while SHIP1-related modulation has been connected to amyloid-β-mediated microglial responses and TREM2-induced microglial functions [23,24,25]. These findings suggest that SHIP1 affects the handling of pathological substrates, although the direction and magnitude of the effect probably depend on the receptor pathway engaged and the intracellular state of the cell.

Despite emerging evidence that uptake and intracellular degradation can be dissociated, these processes, together with inflammatory resolution, are still rarely assessed in parallel. Increased uptake of amyloid-β or lipid-rich material may appear beneficial in plaque assays, but inefficient endosomal maturation, defective lysosomal acidification, or impaired autophagic flux can convert increased phagocytic input into cellular stress [35,37,38,56]. NLRP3 activation provides a plausible downstream consequence of this failure, linking lysosomal damage and mitochondrial stress to caspase-1 activation, IL-1β/IL-18 maturation, and sustained inflammatory signaling [40,41,57]. Future studies should test whether INPP5D/SHIP1 changes substrate selectivity, vesicular routing, lysosomal degradation, and cytokine output in parallel. Without these readouts, an increase in phagocytic signal is difficult to interpret. The same assay may reflect productive amyloid handling, stalled lysosomal delivery, or inappropriate engulfment of vulnerable synaptic structures.

## 4. INPP5D/SHIP1, Endolysosomal Stress, Autophagy, and Inflammasome Activation

Phagocytosis becomes protective only when internalized material can be delivered to degradative compartments and cleared. This requirement is especially demanding in AD, where microglia are chronically exposed to amyloid-β aggregates, lipid-rich debris, damaged synaptic elements, apoptotic material, and inflammatory mediators. Endosomal and lysosomal abnormalities are well documented in AD, and defective autophagy has been implicated in the accumulation of aggregated proteins and damaged organelles [36,37,58]. In microglia, these defects are likely to be amplified by persistent phagocytic input. Retromer dysfunction, impaired lysosomal acidification, and altered autophagic flux can shift microglia from degradative clearance toward intracellular stress [35,37,38,59,60]. These degradative pathways operate within a broader proteostasis network that also includes molecular chaperones. HSP70- and HSP90-associated chaperone systems regulate the folding, stabilization, and disposal of aggregation-prone proteins, whereas chaperone-mediated autophagy provides a selective route for lysosomal degradation of susceptible cytosolic substrates. Dysregulation of these systems has been implicated in AD-associated proteostatic failure, although their direct mechanistic relationship with INPP5D/SHIP1 in microglia remains poorly defined [61,62].

Against this broader proteostatic background, INPP5D/SHIP1 provides a plausible link between phosphoinositide signaling and the endolysosomal machinery. SHIP1 controls the conversion of PIP3 to PI(3,4)P2, and these lipids regulate membrane identity, actin dynamics, endocytosis, and vesicle maturation [9,10,13,14]. Altered SHIP1 activity could change how engulfed amyloid-β, lipids, or cellular debris are sorted after uptake: recycled, retained in endosomal compartments, or delivered to lysosomes. The available AD studies support this connection but do not yet define each trafficking step. INPP5D deficiency attenuates amyloid pathology in mouse models, while SHIP1 modulation has been linked to altered microglial responses to amyloid-β and TREM2-related functions [23,24,25]. More directly, reduced SHIP1 activity in human microglia has been associated with disrupted autophagy and increased inflammasome activation [21,22]. These observations suggest that SHIP1 affects not only substrate uptake, but also the intracellular fate of engulfed material.

Endolysosomal failure provides a route from altered phagocytosis to inflammatory injury. Poorly degraded amyloid-β and lipid-rich material can destabilize lysosomal membranes, promote cathepsin release, increase oxidative and mitochondrial stress, and activate NLRP3 inflammasome signaling [40,41,42,63]. Once engaged, the NLRP3 pathway promotes caspase-1 activation and maturation of IL-1β and IL-18, reinforcing microglial inflammatory output [57,64,65,66]. In parallel, the recently identified INPP5D–RIPK1 interaction provides a distinct mechanism through which SHIP1 can restrain microglial inflammatory programs, including RIPK1-dependent transcriptional induction of NLRP3 and gasdermin D [52]. Together, these findings indicate that the inflammatory consequences of altered SHIP1 activity may arise through both endolysosomal stress and more direct regulation of inflammatory signaling. Lowering SHIP1 activity may enhance selected microglial responses to pathological substrates in some AD settings, but any benefit will depend on whether lysosomal and autophagic capacity can accommodate the resulting cargo load [23,24,25,58,67]. If lysosomal acidification or autophagic flux is compromised, additional cargo input may create a degradative bottleneck, increasing the likelihood of lysosomal damage and inflammasome activation. Future work should measure uptake, vesicular routing, lysosomal acidification, autophagic flux, and cytokine release in the same experimental systems, rather than treating phagocytosis and inflammation as separate outcomes.

## 5. Crosstalk with Other AD Risk Pathways: TREM2, CD33, Complement, and Lipid Metabolism

Several AD risk pathways intersect with INPP5D at the level of microglial receptor signaling and substrate handling. TREM2 is the clearest example. Rare TREM2 variants increase AD risk, and TREM2-dependent programs support microglial clustering around plaques, lipid sensing, cell survival, and metabolic adaptation [7,8,26,27,28,29,30]. Mechanistically, TREM2-DAP12 signaling engages PI3K-related pathways and cytoskeletal remodeling, both of which are sensitive to phosphoinositide composition [16]. SHIP1 sits downstream of this signaling environment by converting PIP3 to PI(3,4)P2. Experimental work showing that SHIP inhibition mediates selected TREM2-induced microglial functions provides a direct link between the two pathways [25]. This does not mean that INPP5D simply blocks TREM2. A more likely model is that SHIP1 tunes how TREM2-related signals are translated into membrane remodeling, phagocytosis, vesicle trafficking, and inflammatory output.

CD33 points to a different side of microglial regulation. As an inhibitory immune receptor associated with AD risk, CD33 has been linked to reduced microglial uptake of amyloid-β and altered monocyte function [31,32]. SHIP1 contains an SH2 domain and is classically involved in signaling downstream of immunoreceptor-associated motifs, giving INPP5D a plausible role in inhibitory receptor programs [9,10,11,12]. In AD, microglia receive simultaneous activating and inhibitory cues from amyloid, lipids, complement, apoptotic material, and cytokines. Changes in SHIP1 activity could shift the threshold at which microglia move from restrained surveillance to phagocytic activation, or from adaptive activation to inflammatory persistence. This remains experimentally underdefined, but it is testable: CD33-related suppression of amyloid uptake, TREM2-related activation, and SHIP1-dependent phosphoinositide remodeling should be examined in the same cellular systems.

**Figure 1:**
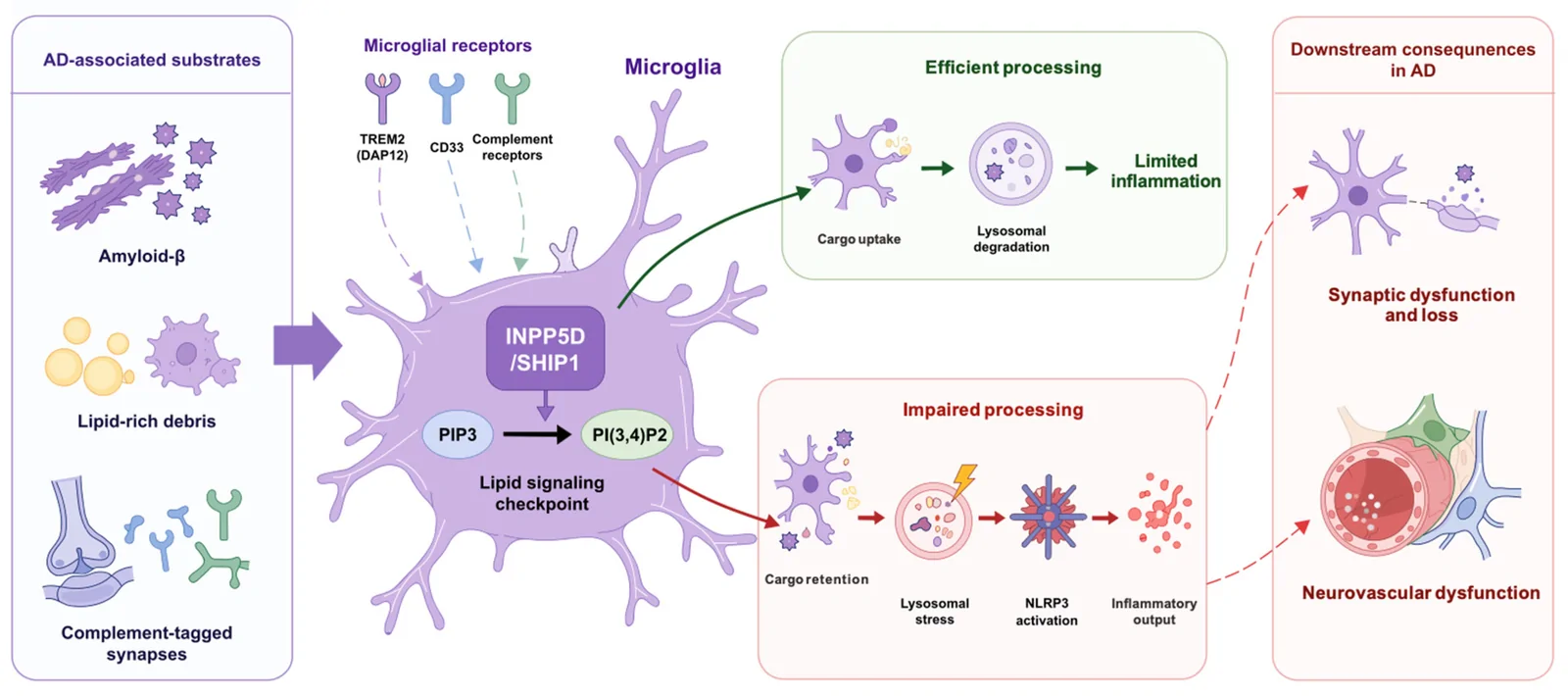
Working model of INPP5D/SHIP1-mediated regulation of microglial substrate handling in Alzheimer’s disease (AD). In the AD brain, microglia are exposed to amyloid-β (Aβ) aggregates, lipid-rich material derived from myelin and apoptotic cells, and complement-tagged synaptic elements. These substrates engage receptor pathways including TREM2–DAP12, CD33, and complement receptors, which converge on phosphoinositide-dependent signaling. INPP5D encodes the lipid phosphatase SHIP1, which converts phosphatidylinositol-3,4,5-trisphosphate (PIP3) to phosphatidylinositol-3,4-bisphosphate [PI(3,4)P2] and thereby modifies membrane-associated signaling, phagocytic uptake, and intracellular cargo routing. Efficient delivery of internalized material to lysosomes supports substrate degradation and limits inflammatory output. By contrast, defective vesicular trafficking or insufficient degradative capacity promotes cargo retention, lysosomal stress, and activation of the NLRP3 inflammasome, followed by inflammatory output. These responses may contribute to synaptic dysfunction and loss, persistent neuroinflammation, and secondary neurovascular dysfunction, including blood–brain barrier dysfunction and vascular inflammation.

Complement and lipid metabolism broaden this network beyond amyloid uptake. Complement proteins can tag synaptic elements for microglial engulfment, and excessive complement-dependent pruning contributes to early synapse loss in AD models [33,34]. Recent work showing that microglial SHIP1 limits complement-mediated synaptic pruning in the developing hippocampus suggests that INPP5D-dependent signaling may also shape complement-driven engulfment programs [68]. These complement-dependent processes also provide a mechanistic interface between microglial substrate handling and glia–neuron communication. Changes in microglial recognition and engulfment of complement-tagged synaptic elements may therefore influence synaptic maintenance and neuronal circuit integrity, in addition to determining substrate clearance. Lipid handling and extracellular proteostasis add another layer of pressure. APOE, ABCA7, TREM2, and CLU intersect with pathways involved in lipid transport, extracellular protein handling, and microglial responses to chronic substrate burden [29,30,69,70,71,72,73,74]. Of particular relevance, CLU functions as an extracellular chaperone that interacts with aggregation-prone proteins, including Aβ, and emerging evidence links CLU-dependent signaling to astrocyte reactivity, complement regulation, microglial phagocytosis, and synaptic integrity [75]. In the AD brain, myelin fragments, apoptotic membranes, lipoprotein particles, and amyloid-associated debris create a sustained lipid burden for microglia. It remains unclear whether SHIP1-dependent signaling influences the fate of these lipid-rich substrates by favoring degradation, intracellular retention, or coupling to inflammatory responses [21,22,35,37,76]. A more useful framework places these pathways within a common cellular workflow, spanning receptor engagement, cargo uptake, endosomal routing, lipid degradation, complement-associated engulfment, and inflammasome priming. Rather than acting as independent risk pathways, they converge on shared mechanisms that determine how microglia process disease-associated substrates. These points of convergence are summarized in Table 1. Integrating these observations further supports a working model in which INPP5D/SHIP1 couples phosphoinositide signaling to substrate handling, lysosomal adaptation, and downstream inflammatory responses in AD microglia (Figure 1).

## 6. Neurovascular Implications of INPP5D-Dependent Microglial Signaling

The neurovascular unit provides the structural and functional interface between the circulation and the brain parenchyma. Endothelial cells, pericytes, astrocytic endfeet, neurons, microglia, perivascular macrophages, and the basement membrane jointly regulate blood–brain barrier integrity, vascular tone, metabolic exchange, immune surveillance, and perivascular clearance [43,44,45,46]. In AD, this system is progressively compromised. BBB breakdown, pericyte dysfunction, altered cerebral blood flow, and impaired amyloid-β clearance have all been associated with cognitive decline, amyloid burden, or neurodegenerative progression [47,48,49]. These vascular abnormalities do not replace amyloid, tau, or microglial mechanisms; they provide a permissive environment in which metabolic stress, inflammatory signaling, and defective clearance can reinforce one another.

At present, the neurovascular relevance of INPP5D is better framed through microglial state changes than through a direct endothelial mechanism. During AD progression, microglia respond to amyloid-β, lipid-rich debris, damaged synapses, complement-tagged structures, and inflammatory mediators [26,27,29,33,34]. If altered SHIP1 activity disrupts substrate handling, lysosomal function, or inflammasome control, the resulting inflammatory state could influence neighboring vascular and perivascular cells [21,22,40,41]. IL-1β, TNF-α, IL-6, reactive oxygen species, and complement-related signals can promote endothelial activation, weaken barrier properties, and alter perivascular inflammatory tone [43,44,45]. Impaired degradation of amyloid-β or lipid-rich material may also increase pathological substrate burden near vascular and perivascular compartments, where vascular clearance routes are already vulnerable in aging and AD [47,48,49,72,74].

The available evidence therefore favors a non-cell-autonomous neurovascular hypothesis rather than an endothelial-autonomous role for INPP5D in AD. Available expression and functional evidence points primarily to a microglial or myeloid-lineage role for INPP5D [17,18,19,20]. Under this model, INPP5D-dependent changes in microglial lipid signaling, phagocytosis, autophagy, inflammasome activation, and cytokine production may alter the local vascular environment. Relevant readouts include perivascular microglial activation, BBB permeability, endothelial inflammatory markers, pericyte coverage, vascular amyloid deposition, cerebral perfusion, and perivascular clearance. This approach keeps the vascular argument testable without overstating direct endothelial involvement.

## 7. Therapeutic Implications: Should SHIP1 Be Inhibited or Enhanced?

Therapeutic targeting of SHIP1 is complicated by its position in the microglial workflow. The same enzyme links phosphoinositide signaling and immune receptor output to membrane remodeling, phagocytosis, vesicle trafficking, lysosomal processing, and inflammatory activation [11,13,14,15,16]. This breadth explains the therapeutic interest in SHIP1, but it also limits simple prediction. In amyloid models, INPP5D deficiency or SHIP1-related modulation has been associated with reduced amyloid pathology and altered plaque-associated microglial responses [23,24]. SHIP inhibition can also mediate selected TREM2-induced microglial functions [25]. These findings support the idea that lowering SHIP1 activity may enhance selected clearance-associated programs, especially when microglia retain sufficient degradative capacity.

The same intervention could become detrimental in a different cellular state. Increased substrate uptake is useful only if internalized material is properly trafficked and degraded. When lysosomal acidification or autophagic flux is impaired, additional phagocytic input may worsen endolysosomal stress [37,38,56]. This concern is not theoretical: reduced SHIP1 activity in human microglia has been linked to disrupted autophagy and enhanced inflammasome activation [21,22], and NLRP3 activation is a well-established driver of amyloid- and tau-associated inflammatory pathology [40,41,42,63]. Preserving or enhancing SHIP1 activity might restrain excessive PI3K-dependent immune activation and cytokine production, but excessive restraint could blunt amyloid-β uptake, lipid handling, apoptotic cell removal, or adaptive plaque-associated responses [26,29,30,31,72]. The therapeutic window is therefore likely to depend on disease stage, amyloid and tau burden, microglial state, lysosomal reserve, APOE genotype, vascular comorbidity, and treatment duration.

A practical therapeutic strategy should be built around microglial state rather than around the assumption that SHIP1 is uniformly protective or harmful. A testable possibility is that early disease stages with insufficient plaque containment may be more compatible with partial, temporally restricted SHIP1 inhibition, whereas later inflammatory stages with lysosomal failure and NLRP3 activation may require a more cautious approach, potentially including preservation of SHIP1-dependent restraint or combination with lysosome- and autophagy-supporting interventions. Cell specificity is equally important. Systemic SHIP1 modulation will affect peripheral immune cells as well as brain-resident microglia, complicating dose selection and safety interpretation [9,10,11,12]. Future studies should define how AD-associated INPP5D variants alter SHIP1 enzymatic activity, phosphoinositide composition, substrate uptake, lysosomal degradation, cytokine release, and neurovascular inflammatory markers in the same experimental systems. Without such measurements, SHIP1 inhibition or activation would remain a blunt perturbation of microglial state rather than a mechanism-guided therapeutic strategy.

## 8. Future Perspectives and Conclusions

The major unresolved issue is how AD-associated INPP5D variation changes SHIP1 function in microglia. Current evidence links INPP5D expression and genetic variation to AD risk and plaque-associated microglial states [17,18,19,20]. Experimental studies further connect SHIP1 activity to amyloid pathology, TREM2-related microglial functions, autophagy, and inflammasome activation [21,22,23,24,25]. Yet these observations do not establish whether disease-associated variation increases SHIP1 activity, reduces it, alters isoform usage, changes subcellular localization, or modifies coupling to specific receptor pathways. These possibilities are not equivalent. A variant that reduces enzymatic activity may enhance PIP3-dependent signaling, whereas a variant that changes SHIP1 recruitment to receptor complexes or endosomal membranes could produce a different phenotype. The field needs to move from association to mechanism.

Expression-based studies alone will not resolve this problem. Future work should pair SHIP1 enzymatic and phosphoinositide measurements with functional readouts of substrate uptake, endosomal routing, lysosomal acidification, autophagic flux, inflammasome activation, cytokine release, and complement-associated synaptic engulfment. Recent advances in whole-endolysosomal patch-clamp recording provide a complementary approach for directly assessing lysosomal ion-channel activity and organelle physiology. Such measurements could be combined with lysosomal pH, trafficking, and autophagic flux assays to determine whether altered SHIP1 signaling is accompanied by functional changes in endolysosomal ion homeostasis and membrane excitability [77]. These readouts should be tested across disease stage, amyloid and tau burden, APOE genotype, and microglial state. Human iPSC-derived microglia can help define INPP5D variant-specific mechanisms, whereas mouse AD models remain necessary for testing plaque-associated microglial responses and tissue-level outcomes in vivo [21,22,23,24,26]. Complementary vertebrate models may be useful for rapid imaging of vascular and inflammatory phenotypes, but their value will depend on careful cell-type interpretation.

For the vascular dimension, the strongest current model remains indirect: INPP5D-dependent microglial states should be tested against BBB permeability, endothelial inflammatory activation, pericyte coverage, vascular amyloid burden, cerebral perfusion, and perivascular clearance [44,47,48,49]. Together, these considerations place INPP5D/SHIP1 at a mechanistic intersection between immune receptor signaling and the intracellular handling of disease-associated substrates. Its contribution is unlikely to follow a simple protective-versus-pathogenic rule. The next step is to define the threshold at which SHIP1-dependent signaling stops supporting controlled substrate handling and begins to favor lysosomal failure, chronic inflammation, synaptic injury, or neurovascular dysfunction. That threshold is likely to differ by disease stage, genetic background, and microglial metabolic state.

## Figures and Tables

**Table 1:** AD-associated microglial pathways potentially linked to INPP5D/SHIP1-regulated substrate handling.

Pathway or Process	Key Factors	Mechanistic Intersection with INPP5D/SHIP1
TREM2–APOE signaling	TREM2, TYROBP/DAP12, APOE, PLCG2	SHIP1 modulates receptor-proximal phosphoinositide signaling downstream of ITAM-associated pathways, providing a potential checkpoint between TREM2–DAP12 activation and microglial phagocytic and metabolic responses [26,27,28,29,30].
CD33-associated inhibitory signaling	CD33	CD33 and INPP5D converge on inhibitory microglial programs that regulate phagocytic and inflammatory responses; however, a direct CD33–SHIP1 signaling interaction in AD microglia remains incompletely established [11,12,31,32].
Substrate uptake and processing	TREM2, CD22, ABCA7, complement receptors	SHIP1 links phosphoinositide-dependent membrane remodeling to microglial substrate handling, with the CD22–INPP5D axis further implicating this signaling node in the post-engulfment degradation of Aβ [13,14,31,33,34,35,55].
Endolysosomal and autophagic function	ABCA7, APOE, TREM2, autophagy regulators	SHIP1-dependent phosphoinositide remodeling may influence endosomal routing, lysosomal delivery, and autophagic adaptation following substrate uptake [35,36,37,38].
Inflammatory and inflammasome signaling	RIPK1, NLRP3, GSDMD	INPP5D/SHIP1 restrains microglial inflammatory signaling through direct SH2-domain-mediated suppression of RIPK1 kinase activation and through mechanisms linking SHIP1 dysfunction to autophagic impairment and enhanced NLRP3 inflammasome activation [21,22,40,41,52,57].
Complement-mediated synaptic remodeling	C1q, C3, complement receptors	SHIP1-dependent signaling has been implicated in complement-mediated synaptic pruning, supporting a potential role for INPP5D in complement-associated engulfment programs; whether this mechanism operates similarly in AD remains to be established [33,34,68].
Lipid handling	APOE, ABCA7, TREM2, CLU	INPP5D/SHIP1 intersects with APOE-, ABCA7-, and TREM2-associated lipid-response programs at the level of microglial substrate handling, linking phosphoinositide signaling to the cellular response to chronic lipid burden [29,30,69,71,72,74].

## Data Availability

No new data were created or analyzed in this study. Data sharing is not applicable to this article.
